# Mycobacterial genomic DNA from used Xpert MTB/RIF cartridges can be utilised for accurate second-line genotypic drug susceptibility testing and spoligotyping

**DOI:** 10.1038/s41598-017-14385-x

**Published:** 2017-11-01

**Authors:** Rouxjeane Venter, Brigitta Derendinger, Margaretha de Vos, Samantha Pillay, Tanya Dolby, John Simpson, Natasha Kitchin, Ashley Ruiters, Paul D. van Helden, Robin M. Warren, Grant Theron

**Affiliations:** 1 DST/NRF Centre of Excellence for Biomedical Tuberculosis Research, SA MRC Centre for Tuberculosis Research, Division of Molecular Biology and Human Genetics, Faculty of Medicine and Health Sciences, Stellenbosch University, Cape Town, South Africa; 2 National Health Laboratory Services, Cape Town, South Africa

## Abstract

Xpert MTB/RIF (Xpert) is a widely-used test for tuberculosis (TB) and rifampicin-resistance. Second-line drug susceptibility testing (DST), which is recommended by policymakers, typically requires additional specimen collection that delays effective treatment initiation. We examined whether cartridge extract (CE) from used Xpert TB-positive cartridges was, without downstream DNA extraction or purification, suitable for both genotypic DST (MTBDR*plus*, MTBDR*sl*), which may permit patients to rapidly receive a XDR-TB diagnosis from a single specimen, and spoligotyping, which could facilitate routine genotyping. To determine the limit-of-detection and diagnostic accuracy, CEs from dilution series of drug-susceptible and -resistant bacilli were tested (MTBDR*plus*, MTBDR*sl*). Xpert TB-positive patient sputa CEs (n = 85) were tested (56 Xpert-rifampicin-susceptible, MTBDR*plus* and MTBDR*sl*; 29 Xpert-rifampicin-resistant, MTBDR*sl*). Spoligotyping was done on CEs from dilution series and patient sputa (n = 10). MTBDR*plus* had high non-valid result rates. MTBDR*sl* on CEs from dilutions ≥10^3^CFU/ml (C_T_ ≤ 24, >“low” Xpert semiquantitation category) was accurate, had low indeterminate rates and, on CE from sputa, highly concordant with MTBDR*sl* isolate results. CE spoligotyping results from dilutions ≥10^3^CFU/ml and sputa were correct. MTBDR*sl* and spoligotyping on CE are thus highly feasible. These findings reduce the need for additional specimen collection and culture, for which capacity is limited in high-burden countries, and have implications for diagnostic laboratories and TB molecular epidemiology.

## Introduction

Of the 10.4 million individuals with active tuberculosis (TB) in 2015, 580 000 were rifampicin (RIF) resistant or multidrug-resistant (MDR), defined as resistance to isoniazid (INH) and RIF^1^. Only ~20% of MDR-TB cases were diagnosed and started on treatment, and only half started on treatment were cured^1^. Extensively drug-resistant (XDR)-TB, which is MDR with resistance to a fluoroquinolone (FQ) and a second-line injectable drug (SLID) comprises 10% of MDR-TB cases, yet is even more underdiagnosed than MDR-TB, very costly to treat, and represents an emerging public health emergency^2–6^.

Xpert MTB/RIF (Xpert) (Cepheid, United States) is a Food and Drug Administration and World Health Organization (WHO)-endorsed nucleic acid amplification test (NAAT) that rapidly detects *Mycobacterium tuberculosis* complex-DNA and RIF-resistance directly from sputa^7–9^. Over 25 million Xpert MTB/RIF cartridges have been consumed and over 30 000 test modules are installed worldwide^10^. The WHO and several national programmes recommend that if Xpert detects resistance, an additional sputum is collected for further drug susceptibility testing (DST) using line probe assays (LPAs), such as MTBDR*plus* (RIF and INH) and MTBDR*sl* (FQs and SLIDs), or phenotypic testing^1,9,11,12^.

Patients, however, often do not rapidly return to the clinic to give another sputum or receive DST results. For example, a study in South Africa found that, even after MTBDR*plus* roll-out, time-to-treatment since initial diagnosis was unacceptably long (~55 days), and that this was partly due to challenges with patient loss-to-follow-up^13^. Furthermore, many patients do not produce sufficient sputum of adequate quality, especially in settings with high rates of HIV^14–17^.

MTBDR*plus* and MTBDR*sl* have suboptimal sensitivity on specimens, and culture is often required prior to DNA extraction and further genotypic testing. Not only can this cause diagnostic delay, but many high burden countries lack the necessary biosafety and laboratory infrastructure for mycobacterial culture and DNA extraction^18–21^. Furthermore, culture can result in the loss of potentially clinically-meaningful resistance^22^. There is hence a need to reduce delays in the diagnosis of drug-resistant TB and use rapid methods that minimise reliance on culture through the direct testing of specimens^23^.

Poor adherence to diagnostic algorithms using MTBDR*plus* and MTBDR*sl* has been reported^5,24,25^. For example, in South Africa, 34% of Xpert RIF-resistant patients failed to receive MTBDR*plus* and, of those confirmed to have MDR-TB, 28% did not receive second-line DST with MTBDR*sl* – despite both LPAs being mandated by the national programme^21^. Novel approaches to reduce this gap in the TB care cascade, which is worsened by the requirement for extra patient visits and additional specimen collection, is a major research priority^26,27^. If TB-testing and first- and second-line DST were possible on the first available specimen, fewer patients would potentially be lost and patients could be diagnosed earlier. This could result in earlier effective treatment initiation, fewer patient- and health systems-costs, and better long-term clinical outcomes.

We therefore conducted a proof-of-concept evaluation on whether *M. tuberculosis*-complex genomic DNA in the PCR-reaction mix from used Xpert cartridges (cartridge extract; CE) - that would otherwise be discarded - was detectable in an accurate manner using MTBDR*plus* and MTBDR*sl*. The feasibility of genotyping on CE by spoligotyping was also tested as this would potentially be useful for research laboratories and programmes seeking to implement routine strain surveillance. We explored the feasibility of Sanger sequencing on CE, as this may be useful for additional genotypic DST. Critically, we evaluated CE for all tests without additional downstream DNA extraction or purification, as not only would extraction require equipment not readily available in routine diagnostic laboratories in high burden settings, but it would complicate laboratory workflows and reduce the attractiveness of our approach. If the CE approach was feasible, it would mean that many laboratories would already have instrumentation available for mycobacterial genomic DNA extraction in the form of GeneXpert^10^ and not need to procure new equipment.

## Material and Methods

### Ethics statement

Methods and protocols were carried out in accordance with relevant guidelines and regulations. The study was approved by the Health Research Ethics Committee of Stellenbosch University (N09/11/296) and the City of Cape Town (#10570). Permission was granted to use anonymised residual specimens collected as part of routine diagnostic practice and thus informed consent was waived.

### Xpert MTB/RIF on dilution series of drug-susceptible- and drug-resistant bacilli

A triplicate tenfold dilution series was made using phenotypically-confirmed drug-susceptible (DS)-TB, MDR-TB and XDR-TB clinical isolates (0–10^6^ CFU/ml) in phosphate buffer (33 mM Na_2_HPO_4,_ 33 mM KH_2_PO_4_; pH 6.8) with 0.025% Tween80 (Sigma-Aldrich, United States). Colony counts were done by plating on 7H11 Middlebrook agar (BD Biosciences, United States). Dilutions containing bacilli (1 ml aliquots) were tested by Xpert (54 in total: six dilutions ranging from 10^1^–10^6^ CFU/ml in triplicate for three strains and hence 18 dilutions each for the DS, MDR, and XDR strains) as well as 0 CFU/ml controls in triplicate, according to the manufacturer’s instructions^9^. Used cartridges were stored at 4 °C prior to CE extraction within 24 h and freezing of the CE at −20 °C.

### Xpert MTB/RIF on clinical specimens

Used Xpert*-TB-*positive cartridges done on sputa from people with symptoms suggestive of TB tested as part of the South African national TB diagnostic algorithm were collected between February 2016 and November 2016 from the National Health Laboratory Services (NHLS), a South African National Accreditation System-accredited, quality-assured diagnostics laboratory in Cape Town, South Africa^11^. Cartridges were stored at 4 °C prior to CE extraction within 5 days. Fifty-six Xpert TB-positive, RIF-susceptible cartridges and 29 Xpert-TB-positve RIF-resistant cartridges were collected. When the NHLS did a MGIT 960 liquid culture on sputum from RIF-resistant patients, we collected the isolate [20/29 (69%) had available isolates]. Isolates were not available from Xpert TB-positive, RIF-susceptible specimens as culture is not routinely done in these patients^11,28^.

### Recovery of mycobacterial genomic DNA from used Xpert MTB/RIF cartridges

**Figure 1:**
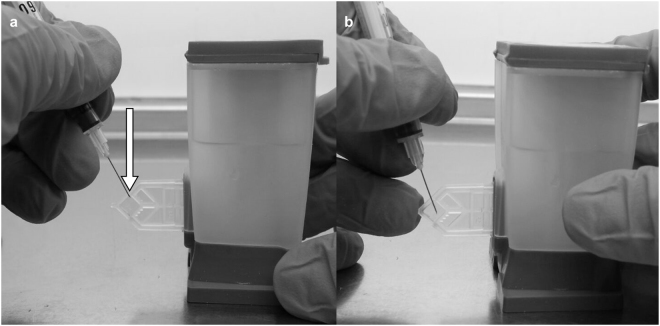
Cartridge extract extraction procedure. (**a**) The arrow indicates the diamond-shaped reaction chamber where the PCR amplification takes place and contains cartridge extract with mycobacterial genomic DNA. The needle is placed at the top of the diamond and the film is slowly and carefully pierced. (**b**) The needle is then slowly inserted deeper into the pocket and cartridge extract mix drawn out without piercing the other side.

The transparent diamond-shaped reaction chamber on the back of the cartridge was punctured with a sterile fixed-needle insulin syringe (1 ml; 29 G) (Fig. 1) in a biosafety level 2 cabinet. The full CE volume, typically ~15 µl, was withdrawn and stored in sterile, safe-lock micro-centrifuge tubes at −20 °C prior to analysis. Each cartridge and the surrounding surface was wiped down thoroughly with 1% sodium hypochlorite and 70% EtOH before and after extraction and UV sterilization was done after each batch of extraction. Used needles were discarded in a sharps container containing 1% sodium hypochlorite. Before and after each cartridge extraction session, hood surface area was decontaminated with sodium hypochlorite and EtOH and UV sterilised. No DNA extraction or purification steps were done on CE.

### Line probe assays on cartridge extract

MTBDR*plus* and MTBDR*sl* (both version 2.0) were done according to the manufacturer’s instructions^29,30^ except for Xpert TB-positive, RIF-susceptible clinical specimens CE (n = 56), 7.5 µl CE was used as imput volume into MTBDR*plus* and MTBDR*sl*. For the Xpert TB-positive, RIF-resistant clinical specimen CEs (n = 29) and the dilution series, 5 µl (the recommended imput volume) CE was used in order to have enough CE remaining for Sanger sequencing. MTBDR*plus* and MTBDR*sl* results were reported as susceptible or resistant (RIF and INH for MTBDR*plus*; FQ and SLID for MTBDR*sl*), indeterminate [*M. tuberculosis* complex DNA*-*positive (reported by the test as TUB-positive) but no gene loci control bands] or TUB-band negative. LPA strips were read by two independent, experienced readers blinded to each other’s calls and Xpert results (and, for dilution series, the strain used).

### Spoligotyping on cartridge extract

Spoligotyping was done as described^31,32^ on 2 µl CE from the MDR-TB dilution series. A set of Xpert TB-positive, RIF-susceptible cartridges (n = 10) done on specimens and separate from those used for genotypic DST on CE were collected with paired culture isolates from an ongoing research study. To determine whether the correct spoligotype was obtained from CE, crude DNA extracted through heat inactivation from the corresponding culture isolates was spoligotyped. SITVIT was used to identify strain families^33^.

### Targeted Sanger sequencing on cartridge extract

For dilution series, PCR clean-up and Sanger sequencing on 5 µl CE was done by the Stellenbosch University Central Analytical Facility using primers overlapping with LPA-binding sites (Supplementary Table 1). The *gyrA* and *rrs* regions in the DS-TB and XDR-TB strains were sequenced.

### Data availability

The datasets generated during and/or analysed during the current study are available from the corresponding author on reasonable request.

## Results

### Patient characteristics

A summary of the patient demographic and clinical data is in Table 1. For Xpert TB-positive, RIF-susceptible patients the median age (IQR) was 40 (31–49) years and for RIF-resistant specimens was 35 (23–42) years. 37/55 (67%) of RIF-susceptible patients and 12/20 (60%) of RIF-resistant patients were male.

**Table 1:** Patient demographic and clinical data.

Patient Characteristics	Xpert TB-positive	
	Xpert rifampicin-susceptible (n = 56)	Xpert rifampicin -resistant (n = 29)
Age, median (IQR)	40 (30–49)	35 (23–42; p = 0.086)
Male gender (%)	37/55 (67)^*^	12/20 (60)*
Smear-positivity (%)	37/50 (74)^*^	6/16 (38)*
Culture-positivity (%)	Not done	19/21 (90)*
TTP, median (IQR)	N/A	10 (8–20)
Xpert C_T_, median IQR	17.9 (16.3–22.1)	20.5 (16.9–24.8)

^*^Missing data: Gender (n = 1 for RIF-susceptible, n = 9 for RIF-resistant); Smear status (n = 6 for Xpert RIF susceptible, n = 13 for Xpert RIF-resistant); Culture results (n = 8 for RIF-resistant results). Abbreviations: Xpert - Xpert MTB/RIF; IQR - interquartile range; TTP - time-to-positivity; C_T_ - cycle threshold values.

### Feasibility and diagnostic accuracy of MTBDR*plus* and MTBDR*sl* on dilution series Xpert TB-positive cartridge extract

**Figure 2:**
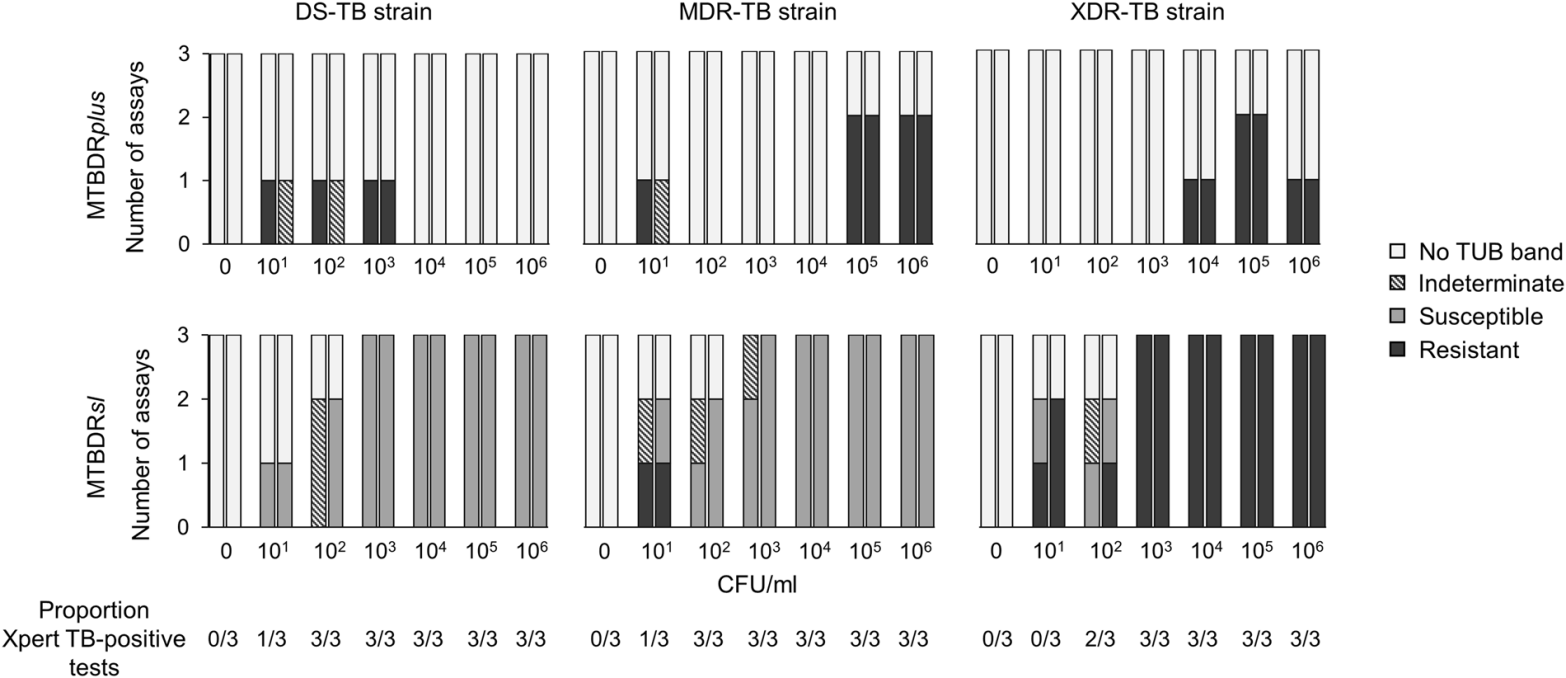
Results of MTBDR*plus* and MTBDR*sl* on Xpert CE from a dilution series of DS-, MDR- and XDR-TB strains. MTBDR*plus* (irrespective of concentration and strain) had high TUB-band negativity and indeterminate rates. However, MTBDR*sl* had high sensitivity and specificity and low indeterminate rates. For each dilution, left bars are for rifampicin (MTBDR*plus*, top row) or fluoroquinolones (MTBDR*sl*, bottom row) and right bars are for isoniazid (MTBDR*plus*) or second-line injectables (MTBDR*sl*). Data from LPA on DS-TB, MDR-TB and XDR-TB strains are shown. The experiment was done in triplicate. Abbreviations: CFU – colony forming; DS-TB – drug susceptible TB; MDR-TB – multidrug resistant TB; XDR-TB – extensively drug resistant TB; units; Xpert - Xpert MTB/RIF.

**Figure 3:**
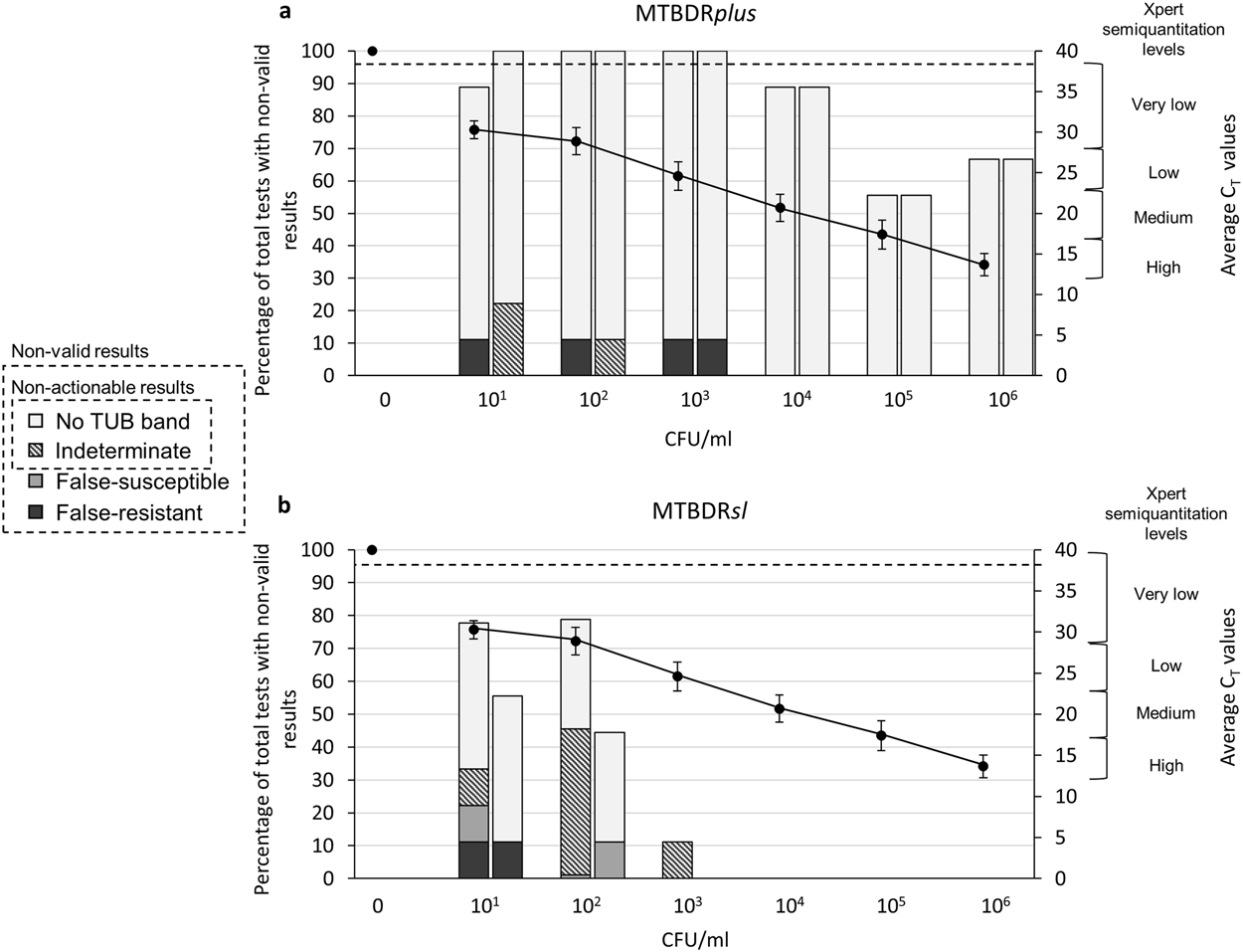
Xpert MTB/RIF quantitative information [average cycle threshold (C_T_) values] (line graph, right y-axes) versus bacterial load (CFU/ml) in a triplicate dilution series for MTBDR*plus* (**a**) and MTBDR*sl* (**b**) done on CE. Left y-axes (bars) show the proportion of assays with non-valid results, disaggregated into non-actionable (TUB-band negative, indeterminate) and non-valid (false-susceptible, false-resistant). For each dilution, left bars are for rifampicin (MTBDR*plus*, top) or fluoroquinolones (MTBDR*sl*, bottom) and right bars are for isoniazid (MTBDR*plus*) or second-line injectables (MTBDR*sl*). Beyond 10^3^ CFU/ml, there were no false resistance or susceptibility calls for MTBDR*sl*, which corresponds to C_T_ ≤ 24. C_T_ ≥ 38 (horizontal dashed line) correspond to a negative Xpert. Error bars show standard error (SE) of average C_T_. Right y-axes show C_T_ corresponding to Xpert semiquantitation levels of very low (C_T_ > 28), low (C_T_ = 22–28), medium (C_T_ = 16–22) and high (C_T_ < 16). Pooled data from LPAs on DS-TB, MDR-TB and XDR-TB strains are shown. Abbreviations: CFU – colony forming; DS-TB – drug susceptible TB; MDR-TB – multidrug resistant TB; XDR-TB – extensively drug resistant TB; CFU – colony forming units; Xpert - Xpert MTB/RIF.

Xpert detected *M. tuberculosis*-complex DNA in all dilutions ≥10^2^ CFU/ml and correctly identified RIF-susceptibility and -resistance (Fig. 2). MTBDR*plus* showed poor overall sensitivity for *M. tuberculosis*-complex DNA [22% (12/54) TUB-band-positive] in CE from Xpert TB-positive cartridges. MTBDR*plus* had high rates of non-actionable (TUB-band negative or TUB-band positive but indeterminate) and false RIF-heteroresistant results (Figs 2 and 3).

In contrast, MTBDR*sl* on CE had high sensitivity and specificity [87% (47/54) and 100% (9/9) respectively] for *M. tuberculosis*-complex DNA and a limit of detection of 10^3^ CFU/ml. Susceptibility and resistance to FQs and SLIDs were correctly detected for all strains ≥10^3^ CFU/ml, corresponding to C_T_ ≤ 24 (the higher C_T_ range of the Xpert “low” semiquantitation category) in all but one sample (one replicate of the MDR-TB strain was indeterminate for FQs; Fig. 3). Once non-actionable results were excluded, overall sensitivities and specificities of 87% (13/15) and 96% (25/26) for FQ-resistance and 94% (15/16) and 97% (30/31) for SLID-resistance, respectively were obtained. When the threshold of ≥10^3^ CFU/ml (C_T_ ≤ 24) was applied, the sensitivity and specificity were both 100% (12/12 and 23/23, respectively) for FQs and for SLIDs (12/12 and 24/24, respectively).

### Diagnostic accuracy of MTBDR*plus* and MTBDR*sl* on clinical specimen Xpert TB-positve cartridge extract

#### Xpert MTB/RIF rifampicin-susceptible specimens

As with the dilution series, MTBDR*plus* had high rates of indeterminate and false-resistance results on clinical specimen CE (Table 2). However, most MTBDR*sl* results from Xpert TB-positive, RIF-susceptible clinical specimen CE were valid (TUB-positive, not indeterminate, and no false-susceptible or -resistant results): 53/56 (95%) for FQ (two TUB-band negative, one indeterminate) and 51/56 (91%) for SLID (two TUB-band negative, three indeterminate). The few CEs that yielded indeterminate MTBDR*sl* results corresponded to “low” or “very low” Xpert semiquantitation levels (C_T_ > 24). The median (IQR) C_T_ of indeterminate (26.3, 24.4–26.7) vs. determinate (17.62, 15.6–20.6) MTBDR*sl* results differed (p < 0.001), indicating that indeterminate results are likely a function of low DNA concentrations in CE. There was not enough CE volume remaining or a matching clinical isolate for confirmatory testing from the three MTBDR*sl*-detected SLIDs resistant patients.

**Table 2:** Results of MTBDR*plus* and MTBDR*sl* drug susceptibility testing using cartridge extract on clinical specimens. MTBDR*plus* had high indeterminate results and rifampicin-resistance false-positive rates. MTBDR*sl* had low indeterminate rates for both DS-TB and DR-TB specimens and performance improved when MTBDR*sl* was done only on specimens with C_T_ ≤ 24.

All Xpert TB-positive specimens						Xpert TB-positive specimens with C_T_ ≤ 24					
Xpert rifampicin-susceptible				Xpert rifampicin-resistant		Xpert rifampicin-susceptible				Xpert rifampicin-resistant	
MTBDR*plus* (n = 56)		MTBDR*sl* (n = 56)		MTBDR*sl* (n = 29)*		MTBDR*plus* (n = 49)		MTBDR*sl* (n = 49)		MTBDR*sl* (n = 20)*	
TUB-band positive (%) 47/56 (84)		TUB-band positive (%) 47/54 (96)		TUB-band positive (%) 27/29 (93)		TUB-band positive (%) 45/49 (92)		TUB-band positive (%) 49/49 (100)		TUB-band positive (%) 20/20 (100)	
**Rifampicin (%)**		**Fluoroquinolones (%)**		**Fluoroquinolones (%)**		**Rifampicin (%)**		**Fluoroquinolones (%)**		**Fluoroquinolones (%)**	
Susceptible	0/47 (0)	Susceptible	53/54 (98)	Susceptible	14/27 (52)	Susceptible	0/56 (0)	Susceptible	49/49 (100)	Susceptible	11/20 (55)
Resistant	47/47 (100)	Resistant	0/54 (0)	Resistant	10/27 (37)	Resistant	45/49 (92)	Resistant	0/49 (0)	Resistant	9/20 (45)
Indeterminate	0/47 (0)	Indeterminate	1/54 (2)	Indeterminate	3/27 (11)	Indeterminate	0/49 (0)	Indeterminate	0/49 (0)	Indeterminate	0/20 (0)
**Isoniazid (%)**		**Second-line injectables %)**		**Second-line injectables (%)**		**Isoniazid (%)**		**Second-line injectables (%)**		**Second-line injectables (%)**	
Susceptible	11/47 (23)	Susceptible	48/54 (88)	Susceptible	15/27 (56)	Susceptible	11/49 (23)	Susceptible	46/49 (94)	Susceptible	14/20 (70)
Resistant	0/47 (0)	Resistant	3/54 (6)	Resistant	9/27 (33)	Resistant	0/49 (0)	Resistant	1/49 (2)	Resistant	6/20 (30)
Indeterminate	36/47 (77)	Indeterminate	3/54 (6)	Indeterminate	3/27 (11)	Indeterminate	34/49 (69)	Indeterminate	2/49 (4)	Indeterminate	0/20 (0)
**TUB-band negative** (%) 9/56 (16)		**TUB-band negative** (%) 2/56 (4)		**TUB-band negative** (%) 2/29 (7)		**TUB-band negative** (%) 4/49 (8)		**TUB-band negative** (%) 0/49 (0)		**TUB-band negative** (%) 0/20 (0)	

*For the 29 Xpert RIF-resistant specimens we were able to retrieve 20 paired culture isolates used for MTBDRsl. 18/20 matched for FQs and 17/20 for SLIDs, the 2/20 done on crude DNA had LPA results whereas the LPA on CE was TUB-band negative. 1/20 did not match for the SLID resistance. Both the TUB-band negative and discordant SLIDs result corresponded to “very low” semi-quantitation level. When defined threshold of C_T_ ≤ 24 was applied all LPAs on CE matched LPA from culture isolates.

#### Xpert MTB/RIF rifampicin-resistant specimens

MTBDR*sl* on Xpert TB-positive, RIF-resistant CE had 24/29 (83%) valid results. For FQs, 14/24 (58%) were susceptible and 10/24 (42%) were resistant. For SLIDs, 15/24 (63%) were susceptible and 9/24 (37%) resistant. The five non-valid results were TUB-band-negative [2/29 (7%)] or indeterminate for both FQs and SLIDs [3/29 (10%); Table 2]. All CEs corresponding to the higher C_T_ ranges of the Xpert “low” semiquantitation category (C_T_ ≤ 24) had valid results, whereas those that had indeterminate or TUB band-negative results corresponded to the lower semiquantitation levels (C_T_ > 25.0). The median (IQR) C_T_ of indeterminate (29.1, 26.5-31.1) vs. determinate (20.5, 16.−23.2) results differed significantly (p < 0.001).

#### MTBDR*plus* and MTBDR*sl* performance on Xpert MTB/RIF cartridge extract by smear status

MTBDR*plus* had high non-valid result rates irrespective of smear status. However, MTBDR*sl* on CE from smear-negative sputums had significantly higher rates of non-actionable results [5/23 (22%) vs. 1/43 (2%) for FQ, p = 0.01; 6/23 (23%) vs. 2/43 (5%) for SLIDs, p = 0.01] compared to smear-positive patients (Supplementary Table 2).

#### Concordance of MTBDR*sl* results on cartridge extract and culture isolates

Of the 29 Xpert TB-positive, RIF-resistant patients, 20 (69%) matched culture isolates were collected while the remaining nine had negative or contaminated cultures. The CEs and isolates showed 18/20 (90%) matching MTBDR*sl* FQ results and 17/20 (84%) matching SLID results. There were 2/20 (10%) discordant TUB-band MTBDR*sl* results on culture isolates (one TUB-positive and FQ and SLID sensitive, one TUB-positive and FQ and SLID resistant) where both CE results were TUB-band negative. There was also 1/20 (5%) discordant SLID result (CE showed resistance but the isolate showed susceptibility). Importantly, all three discordant results corresponded to a “very low” semiquantitation (C_T_ > 28.0). All TUB-band, susceptibility and resistance calls were concordant at C_T_ ≤ 24, indicating that the diagnostic accuracy of MTBDR*sl* on CE vs. isolates is likely comparable at this threshold.

### Spoligotyping on cartridge extract

#### Dilution series

Spoligotyping resulted in a readable strain type for dilutions ≥10^3^ CFU/ml, corresponding to the same threshold seen for MTBDR*sl*.

#### Clinical specimens

Spoligotyping on specimen CE and crude DNA from matched culture isolates were highly concordant 10/10 (100%) at the threshold defined by the dilution series (Table 3). A variety of strain families were observed with Beijing as the predominant family type [6/10 (60%)] as well as 2/10 (20%) LAM and 2/10 (20%) T1 family type.

**Table 3:**
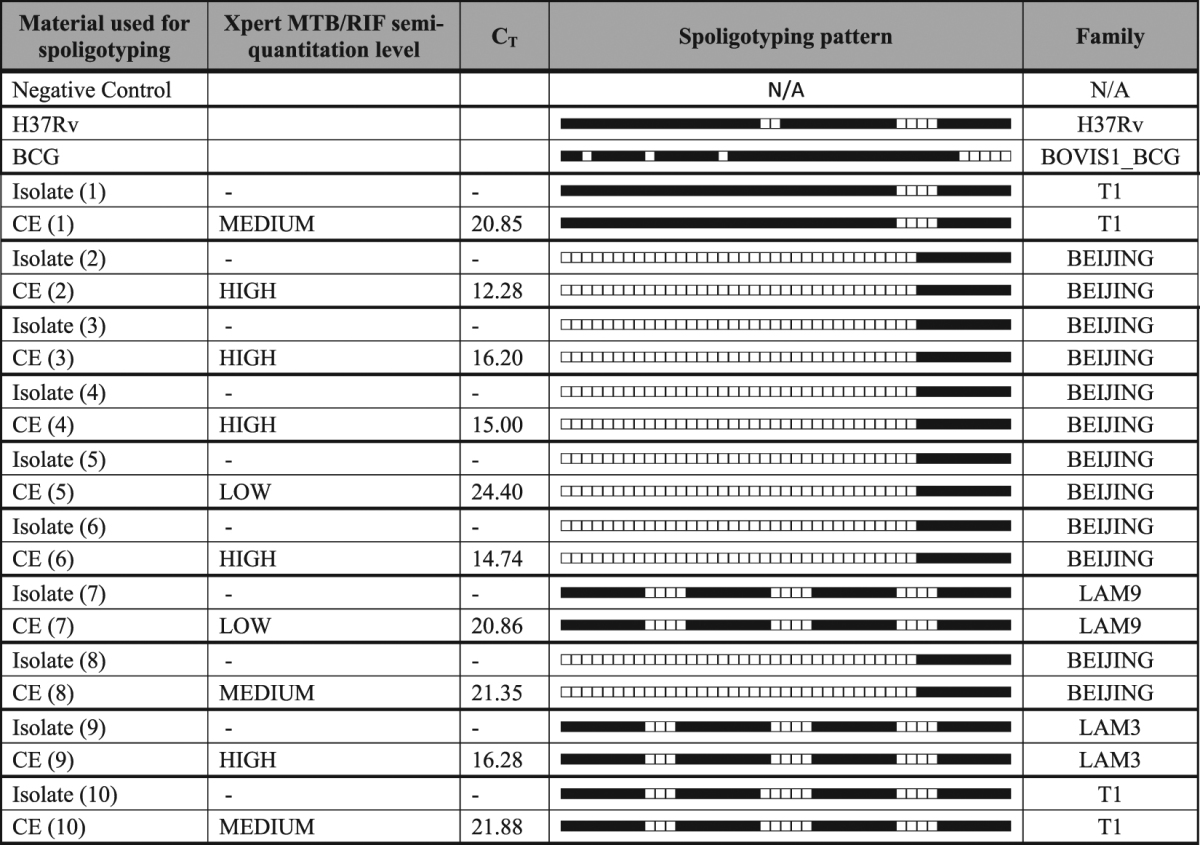
Spoligotyping results performed on CE done on sputum specimens and paired culture isolates at defined threshold (C_T_ ≤ 24).

### Targeted sequencing on extract from used Xpert MTB/RIF cartridges

#### Dilution Series

Targeted Sanger sequencing was done on dilution series CE. For the *rrs* PCR on CE, sequence shorter than the expected length was observed. PCR of *gyrA* from CE from dilutions 10^3^–10^4^ CFU/ml resulted in sequence expected length, however high background noise occurred and the sequence did not align to H37Rv [NC_000962]. *gyrA* on CE from dilutions 10^5^–10^6^ CFU/ml aligned to the reference genome, however, several SNPs known to be present in the resistance determining regions (identified by sequencing of the corresponding isolate) were not detected. Due to the relatively poor limit of detection and accuracy of Sanger sequencing on dilution series CE, we did not do sequencing on clinical specimen CEs.

## Discussion

Our key findings are: (1) MTBDR*sl* on CE enabled genotypic drug-susceptibility testing for FQs and SLIDs with high accuracy and low indeterminate rates when the Xpert semiquantitation category was at least “medium” or C_T_ ≤ 24 (corresponding to ≥ 10^3^ CFU/ml), (2) spoligotyping was feasible and accurate on CE at the same threshold, (3) MTBDR*plus* was not feasible or accurate on CE and (4) neither was Sanger sequencing. These data have implications for the routine diagnosis of drug-resistant TB, researchers, and test developers.

Xpert is one of the most widely used tests for TB and drug-resistance^9,34^ and although it is a significant advancement, time-to-treatment – especially for MDR- and XDR-TB - is still very long^35–38^. Our results show that accurate second-line drug testing using MTBDR*sl* is possible on CE from Xpert cartridges that would otherwise be discarded. This potentially allows for a rapid, single-specimen diagnosis of XDR-TB without additional specimen collection. Importantly, we defined a threshold at which this approach is feasible, meaning that MTBDR*sl* assays do not need to be wasted on CE unlikely to give a valid result. Using this threshold, we showed that on clinical specimen CEs, susceptibility and resistance calls were concordant with those from the isolate^19,39^. Furthermore, we showed that it is possible to do spoligotyping on CE at this threshold, which will inform strain surveillance and research studies on relapse and reinfection where specimens are limited. Collectively, these findings may reduce the need for culture.

Although our data suggest that the MTBDR*sl* will work on CE from cartridges with an Xpert semiquantitation category of at least “low”, we suggest that, in laboratories where C_T_ cannot be readily calculated, a category of at least “medium” is used to guide use of this strategy unless the laboratory is comfortable with some semiquantitation low specimens not having a valid MTBDR*sl* result. Alternatively, if smear microscopy is available, smear-positivity may be used to guide use of CE, however, some smear-negative specimens in whom this approach would work (10^3^-10^4^ CFU/ml) would be unnecessarily excluded.

When considering the CE approach, it is important to identify a safe and sterile environment to avoid contamination. Although Xpert sample reagent as well as the sonication lysis step within the cartridge helps ensure *M. tuberculosis* is no longer culturable (and therefore poses minimal infectious risk^40^), steps to minimise the risk of *rpoB* amplicon cross-contamination should be implemented. These can include working in a dedicated cabinet or room and sterilising the work area with UV and disinfectant after CE is collected. Importantly, however, cross-contamination of other Xpert cartridges with *rpoB* amplicons appears unlikely. Although Xpert’s automated pre-amplification wash step does not remove large pieces of debris-associated genomic DNA, it does efficiently remove high concentrations of contaminating *rpoB* amplicons from assays like MTBDR*plus* ^41,42^. NAATs without such a wash step may be more vulnerable to CE *rpoB* amplicon cross-contamination.

Our study differed from a previous study which showed that sequencing, MTBDR*plus*, spoligotyping and MIRU-VNTR typing are feasible on the sputum mixed with Xpert sample reagent^43^. However, this sample reagent method has a number of disadvantages: 1) often no volume remains, 2) prolonged exposure to sample reagent degrades DNA and potentially introduces mutations^9,40^, and 3) it still requires DNA extraction prior to PCR. Furthermore, DNA extraction adds cost and is not always feasible in laboratories in high burden countries; whereas the CE method yields directly usable material and does not need additional extraction or purification steps. An advantage, however, of using the sputum mixed with Xpert reagent buffer, is that it likely avoids high MTBDR*plus* error rates (TUB-band negative, indeterminate, false-positive) seen with CE. This could be due to the large amount of *rpoB* amplicons in Xpert TB-positive CE, which share binding sites with MTBDR*plus* probes and confound the assay resulting in non-valid results. Furthermore, the *rpoB* PCR that occurs as part of MTBDR*plus* may sequester reagents away from the multiplex *inhA* and *katG* amplification reactions. Testing for mutations conferring INH resistance using CE might hence be possible with the Genoscholar INH II line probe assay (which does not contain *rpoB* probes)^44^. Sequencing from CE thus primarily appears to be driven by *rpoB* amplicon interference (although a PCR clean-up was done prior to sequencing, this would have co-purified *rpoB* amplicons). Further investigation with primers optimised for minimal-input DNA may be warranted, however, it appears that, for sequencing, the best approach to avoid contaminating amplicons might be to PCR from the specimen-Xpert sample reagent mixture^43^. Given the rates of non-valid CE results below the defined threshold, we suggest that specimen-Xpert sample reagent mix be kept in the event that C_T_ falls >24.

The results presented here should be interpreted in context of their limitations. For the clinical specimens tested from the NHLS, matched culture isolates were not available for Xpert RIF-susceptible specimens, as per the national algorithm. However, the dilution series experiments showed very high concordance between MTBDR*sl* on CE vs. the isolates. The utility of CE depends on the downstream test used and MTBDR*sl* susceptible or non-valid results should be interpreted from CE the same as when they are done on patient specimens (i.e., further investigation, including culture, is recommended)^45^. Realistically, cartridges may need to be transported from remote locations and so the effect of storing cartridges for prolonged duration (>5 days) and at ambient temperature requires further systematic testing. Using bacilli in buffer can have limitations, which is why we also used patient clinical specimens, which are a better material to test than bacilli added to sputum (the former has bacilli within a sputum matrix, whereas in the latter bacilli are typically freely floating in bubbles).

In conclusion, CE contains template DNA for second-line DST using MTBDR*sl*, resulting in accurate results highly concordant with those from isolates, provided bacillary load in the specimen corresponds to at least a “medium” Xpert semiquantitation category of C_T_ ≤ 24. This potentially facilitates XDR-TB detection within days from a single specimen. Spoligotyping is also feasible on CE and works consistently at this threshold. Our method provides an opportunity to potentially reduce the burden associated with addition specimen collection, such as patient treatment delay, pre-treatment loss-to-follow-up, and increased patient and provider costs. Furthermore, it shows that material that would otherwise be discarded still holds diagnostic utility.

## Electronic supplementary material

Supplementary information
