# Supplementary material for: Mycobacterial genomic DNA from used Xpert MTB/RIF cartridges can be utilised for accurate second-line genotypic drug susceptibility testing and spoligotyping
Source: Sci Rep. 2017 Nov 1;7:14854. doi: 10.1038/s41598-017-14385-x (PMC5666021; doi:10.1038/s41598-017-14385-x)
Supplement: Supplementary file 1 — Supplementary information [file 41598_2017_14385_MOESM1_ESM.pdf]

Mycobacterial genomic DNA from used Xpert MTB/RIF cartridges can be utilised for accurate second-line genotypic drug susceptibility testing and spoligotyping

Rouxjeane Venter[1,+], Brigitta Derendinger[1,+], Margaretha de Vos[1], Samantha Pillay[1,2], Tanya Dolby[2], John Simpson[2], Natasha Kitchin[1], Ashley Ruiters[2], Paul D. van Helden[1], Robin M. Warren[1], Grant Theron[1,*]

[1]DST/NRF Centre of Excellence for Biomedical Tuberculosis Research, SA MRC Centre for Tuberculosis Research, Division of Molecular Biology and Human Genetics, Faculty of Medicine and Health Sciences, Stellenbosch University, Cape Town, South Africa.
[2]National Health Laboratory Services, Cape Town, South Africa.

+ These authors contributed equally to this work

Supplementary

Table 1: Primer details for amplification of fragments of the *gyrA* and *rss* regions using cartridge extract from the dilution series

**Target region**	**Primer name**	**Primer sequence (5' – 3')**	**Annealing temperature (°C)**	**Expected size (bp)**
gyrA	gyrA_F	TGACATCGAGCAGGAGATGC	62	344
	gyrA_F	GGGCTTCGGTGTACCTCATC		
rrs290	rrs290_F	TGCTACAATGGCCGGTACAA	62	290
	rrs290_R	CTTCCGGTACGGCTACCTTG		

Table 2: Results of MTBDR*plus* and MTBDR*sl* drug susceptibility testing using cartridge extract on clinical specimens stratified to smear status. MTBDR*plus* had high indeterminate results rifampicin-resistance false-positive rates for both smear positive and smear negative specimens. MTBDR*sl* had low indeterminate rates for both RIF-susceptible and RIF–resistant specimens. Smear negative specimens had higher rates of non-actionable results for MTBDR*sl*.

**Xpert positive rifampicin-susceptible and -resistant cartridges***								
**Smear-positive specimens**				**Smear-negative specimens**				
**MTBDR*plus* (n=37)**		**MTBDR*sl* (n=43)**		**MTBDR*plus* (n=13)**		**MTBDR*sl* (n=23)**		**p-values†**
**TUB-band positive 32/37 (86)**		**TUB-band positive 42/43 (98)**		**TUB-band positive 10/13 (77)**		**TUB-band positive 21/23 (91)**		
**Rifampicin** (%)		**Fluoroquinolones** (%)		**Rifampicin** (%)		**Fluoroquinolones** (%)		p=0.01
Susceptible	0/32 (0)	Susceptible	41/42 (98)	Susceptible	0/10 (0)	Susceptible	17/21 (81)	
Resistant	32/32 (100)	Resistant	1/42 (2)	Resistant	10/10 (100)	Resistant	1/21 (5)	
Indeterminate	0/32 (0)	Indeterminate	0/42 (0)	Indeterminate	0/10 (0)	Indeterminate	3/21 (14)	
**Isoniazid** (%)		**Second-line injectables** (%)		**Isoniazid** (%)		**Second-line injectables** (%)		p=0.01
Susceptible	10/32 (31)	Susceptible	39/42 (93)	Susceptible	0/10 (0)	Susceptible	16/21 (76)	
Resistant	0/37 (0)	Resistant	2/42 (5)	Resistant	0/10 (0)	Resistant	1/21 (5)	
Indeterminate	22/32 (69)	Indeterminate	.1/42 (2)	Indeterminate	10/10 (100)	Indeterminate	4/21 (19)	
**TUB-band negative**				**TUB band-negative**				p=0.015
5/37 (14)		1/43 (2)		3/13 (23)		2/23 (9)		

* Table shows results from both Xpert positive RIF-susceptible and RI-resistant specimens. RIF-susceptible samples had MTBDR*plus* and MTBDR*sl* done. RIF-resistant specimens only had MTBDR*sl*. 37/56 (66%) RIF-susceptible specimens were smear positive; 13/56 (23%) were smear negative and 6/56 (11%) had no smear results. 6/29 (20%) of RIF-resistant specimens were smear positive while 10/29 (35%) were smear negative and 13/29 (45%) had no smear result.
